# Resolving the Conflict Between Regional Analgesia and Developing Compartment Syndrome in Below-Knee Surgeries With the High-Volume Proximal Adductor Canal (Hi-PAC) Block: A Prospective Feasibility Study

**DOI:** 10.7759/cureus.23898

**Published:** 2022-04-06

**Authors:** Kartik Sonawane, Ankita Shah, Jagannathan Balavenkatasubramanian

**Affiliations:** 1 Anesthesiology, Ganga Medical Centre and Hospitals Private Limited, Coimbatore, IND; 2 Anesthesiology and Perioperative Medicine, Ganga Medical Centre and Hospitals Private Limited, Coimbatore, IND

**Keywords:** postoperative pain management, procedure-specific ra, foot and ankle surgeries, regional anesthesia, regional analgesia, compartment syndrome, below-knee surgeries

## Abstract

The consideration of regional analgesia (RA) in below-knee surgeries is always a controversial topic due to the fear of masking symptoms of developing compartment syndrome (CS) in the postoperative period. Compartment syndrome (CS) has been found frequently in below-knee surgeries, particularly among tibial diaphyseal fractures. Like any other surgery, below-knee surgeries have significant postoperative pain that requires effective postoperative analgesia protocol. The analgesia quality makes a big difference when compared with or without RA. Also, the presence or absence of RA cannot prevent or promote the development of CS. Therefore, patients should not be deprived of their right to remain pain-free in the postoperative period by compromising the analgesia protocol.

The pain out of proportion to the surgery or injury is a typical symptom of developing CS, which can cause increased analgesic demands postoperatively. Timely diagnosis and treatment of CS require vigilant postoperative monitoring of the warning signs by trained staff. Avoiding RA for fear of presumed masking of symptoms and delaying CS diagnosis may not be a solution instead of choosing an appropriate RA with regular postoperative monitoring for such warning symptoms. The high-volume proximal adductor canal (Hi-PAC) block has been described as a procedure-specific and motor-sparing RA technique appropriate for below-knee surgeries.

In this prospective study, we evaluated the analgesic efficacy of the Hi-PAC block in below-knee surgeries. We also observed the effect of the Hi-PAC block, due to proximal and distal drug distribution, on masking the symptoms of the developing CS during postoperative monitoring.

We found the Hi-PAC block to be a safer and more effective RA alternative for below-knee surgeries with an added motor-sparing benefit that facilitated early mobility and discharge. Its property of not interfering with postoperative surveillance to detect the symptoms of CS and intervene in time helps deal with the anxiety of CS in below-knee surgeries.

## Introduction

Perioperative pain management for patients undergoing below-knee surgeries is challenging due to the concerns about the postoperative development of acute compartment syndrome (ACS). ACS is a true orthopedic/traumatic/surgical emergency that can lead to ischemia and eventual necrosis if left untreated, resulting in significant morbidity and mortality. The incidence of ACS is 1%-10% of all leg fractures, of which 75% of cases are associated with long bone fractures (predominantly the tibial diaphysis), followed by distal radial fractures (18%) [1-3]. It can develop during surgery, in the immediate postoperative period, or up to 72 hours after surgery [4].

Fear or anxiety of ACS deprives patients of the optimal analgesic options and exposes them to severe postoperative pain, resulting in an unsatisfactory hospital stay. Dependency on systemic analgesics alone often fails to meet escalating postoperative demands resulting in inadequate analgesia or oligoanalgesia. A comprehensive team approach is required to manage such a preventable and treatable situation. It requires identifying high-risk patients or surgeries prone to developing ACS, providing the most suitable analgesic regimen, and periodic vigilant monitoring of the signs and symptoms of impending compartment syndrome (CS) postoperatively by trained personnel [5-11].

The application of regional analgesia/anesthesia (RA) in below-knee surgery is contentious due to the belief that it can mask ACS symptoms and put the patient at an unacceptably high risk of serious morbidity. However, evidence for such an assumption is lacking. We believe that RA neither masks the clinical symptoms of impending CS nor prevents ACS development. The development of ACS can be inevitable under favorable circumstances regardless of the presence or absence of RA. Therefore, the mere fear of preventable and treatable conditions such as ACS should not deprive patients of their right to remain pain-free with effective and complete postoperative pain management.

An ideal RA technique for below-knee surgeries should provide effective analgesia without masking the symptoms of the developing CS and facilitate early mobilization and discharge. The high-volume proximal adductor canal (Hi-PAC) block is one such suitable RA technique described for below-knee surgeries [12-14]. In our hospital, the Hi-PAC block has been used postoperatively since 2020 in patients scheduled for below-knee orthopedic surgeries as a component of multimodal analgesia (MMA) protocol. Prior to 2020, the standard of care was systemic analgesics with or without RA techniques such as epidural infusion or a combination of the sciatic nerve (SCN) and saphenous nerve (SN) blocks.

We aimed to observe the analgesic efficacy of the Hi-PAC block along with its technical ease of administration, the clinical effect due to drug distribution pattern, and possible interference during daily postoperative evaluations to detect the symptoms of CS in patients included in this prospective study. We hypothesized that the Hi-PAC block would provide the desired procedure-specific and motor-sparing postoperative analgesia without masking the symptoms of the impending CS. We propose a new paradigm and an alternative RA technique to ensure safer and more efficient postoperative analgesia in below-knee surgeries.

## Materials and methods

This prospective study included 15 patients of either sex with the American Society of Anesthesiologists (ASA) grades I and II who underwent lower extremity orthopedic surgeries below the knee joint under neuraxial anesthesia. We conducted this study from September 2021 to October 2021 with our hospital institutional review board (IRB) approval (reference number IRB/GH/Anaesthesia/008/2021/004). All study patients provided informed consent for anonymous data recording and sharing concerning this study.

Patient preparation

Preoperatively, all patients were premedicated with intravenous pantoprazole 40 mg, ramosetron 0.3 mg, and midazolam 2 mg. The proposed surgery was performed under neuraxial blockade, either spinal or combined spinal-epidural under tourniquet control. Intraoperatively, paracetamol 1 gm, ketorolac 30 mg, and dexamethasone 8 mg were administered intravenously as MMA protocol. The remainder of the intraoperative course of all patients was uneventful, without any need for change in anesthesia or analgesia protocol.

Postoperatively, all patients were monitored in the recovery room and periodically assessed for the recovery of motor movements. In possible cases, preprocedural scans of the femoral nerve (FN) and popliteal SCN were performed to compare sonoanatomy before and after the block. After confirming motor recovery (active toe movements) prior to sensory (pain at the surgical site), the Hi-PAC block was administered using 40 mL of local anesthetic (LA) solution (0.1% ropivacaine + dexamethasone 8 mg).

Ultrasound-guided Hi-PAC block

**Figure 1:**
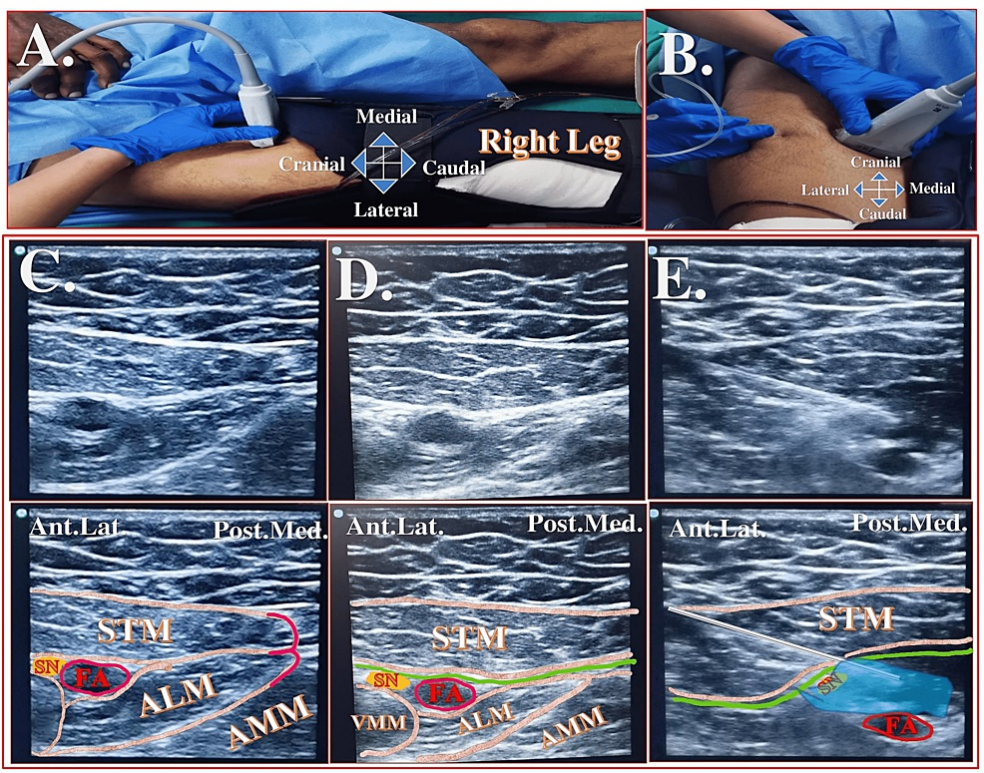
Probe position and relevant sonoanatomy of the high-volume proximal adductor canal (Hi-PAC) block (A) Probe position of the Hi-PAC block. (B) Needle insertion during Hi-PAC block. (C) Sonoanatomy at the level of the apex of the femoral triangle appears as a “figure of 3” formed by the medial borders of the sartorius and the adductor longus muscles. (D) Sonoanatomy at the level of proximal adductor canal (target site). (E) Needle direction and drug deposition during Hi-PAC block. The green line below STM indicates the vasoadductor membrane. The white line indicates the needle. The blue area indicates the drug spread. Hi-PAC: high-volume proximal adductor canal; FA: femoral artery; STM: sartorius muscle; VMM: vastus medialis muscle; ALM: adductor longus muscle; AMM: adductor magnus muscle; SN: saphenous nerve; Ant.Lat.: anterolateral; Post.Med.: posteromedial

A high-frequency linear ultrasound probe (SonoSite HFL38x/13-6 MHz, Fujifilm SonoSite, Bothell, WA, USA) was held transversely over the midthigh with the patient lying supine (Figure 1A). Initially, the apex of the femoral triangle (FT) was identified as the intersection of the medial borders of the adductor longus and sartorius muscles, appearing as a sign of “3” or “kissing sign” under ultrasound (Figure 1C) [13,14]. Then, the ultrasound probe was moved 2-3 cm distal to the apex of the FT to reach the proximal adductor canal (target site) (Figure 1D). A 100-mm block needle (Stimuplex A, B Braun Melsungen, AG, Melsungen, Germany) was inserted in-plane from the lateral to the medial direction between the sartorius and the vastus medialis muscles (Figure 1B). After identifying the hyperechoic SN lateral to the femoral artery (FA), LA solution was deposited around the SN (10 mL) and perivascularly around the FA (30 mL) under the vasoadductor membrane (Figure 1E).

Thirty minutes after the Hi-PAC block, all patients’ toe movements were reevaluated. They were monitored in the recovery room till complete regression of spinal level and attaining discharge criteria. A postoperative MMA protocol was followed: oral paracetamol 1 gm four times a day, aceclofenac 100 mg twice a day, and pregabalin 75 mg at bedtime. Postoperative pain scores were recorded before and after the block and periodically for up to 24 hours using a visual analog scale (VAS) of 0-10 (0 = no pain and 10 = worst pain imaginable). Focused postoperative evaluations to screen the signs and symptoms of ACS were performed daily till the discharge of the patient. All patients were discharged on the fifth postoperative day with a mean pain score of 2/10 on VAS without any requirement of rescue analgesia or opioids.

Primary and secondary outcomes

The primary outcome of this study was to determine the analgesic efficacy of the Hi-PAC block in providing postoperative analgesia for below-knee surgeries. The secondary outcomes included determining the duration to administer the block, determining the extent of the proximal drug distribution to check the involvement of additional neural components (nerve to vastus medialis, SN, and FN), confirming distal drug distribution around the SCN in the popliteal fossa, determining the distance of the drug spread around SCN from the popliteal crease, and calculating the total duration of the analgesia.

Outcome measurement

The measured outcomes included the demographics of the patients, type of neuraxial anesthesia, duration of surgery, vital parameters, time to regain toe movement, time to administer block, proximal and distal drug distribution after block, pain scores (at rest and after squeezing leg muscles) at regular intervals for the first 24 hours, total analgesic duration, presence of any signs of compartment syndrome and complications till discharge.

## Results

**Figure 2:**
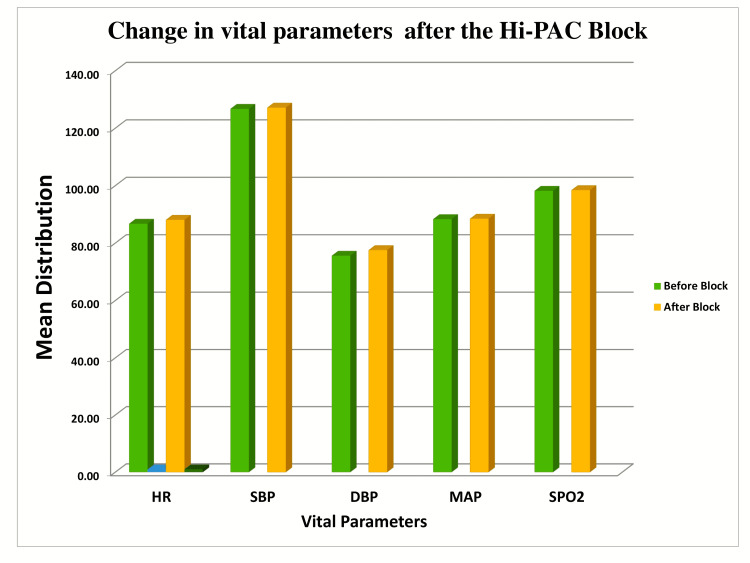
Bar diagram depicting the change in vital parameters before and after the high-volume proximal adductor canal (Hi-PAC) block HR: heart rate; SBP: systolic blood pressure; DBP: diastolic blood pressure; MAP: mean arterial pressure

**Figure 3:**
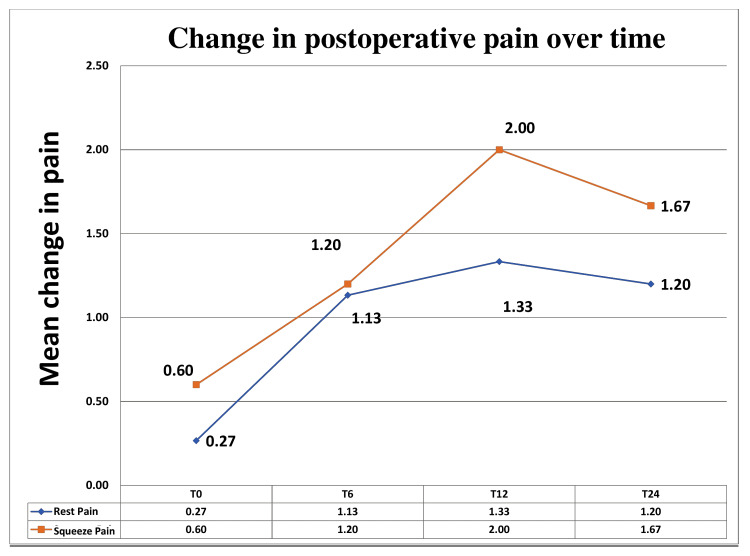
Line diagram depicting the change in postoperative pain scores (at rest and after squeezing) over time Hi-PAC: high-volume proximal adductor canal; T0: pain scores before the Hi-PAC block; T6: pain scores six hours after the Hi-PAC block; T12: pain scores 12 hours after the Hi-PAC block; T24: pain scores 24 hours after the Hi-PAC block

**Figure 4:**
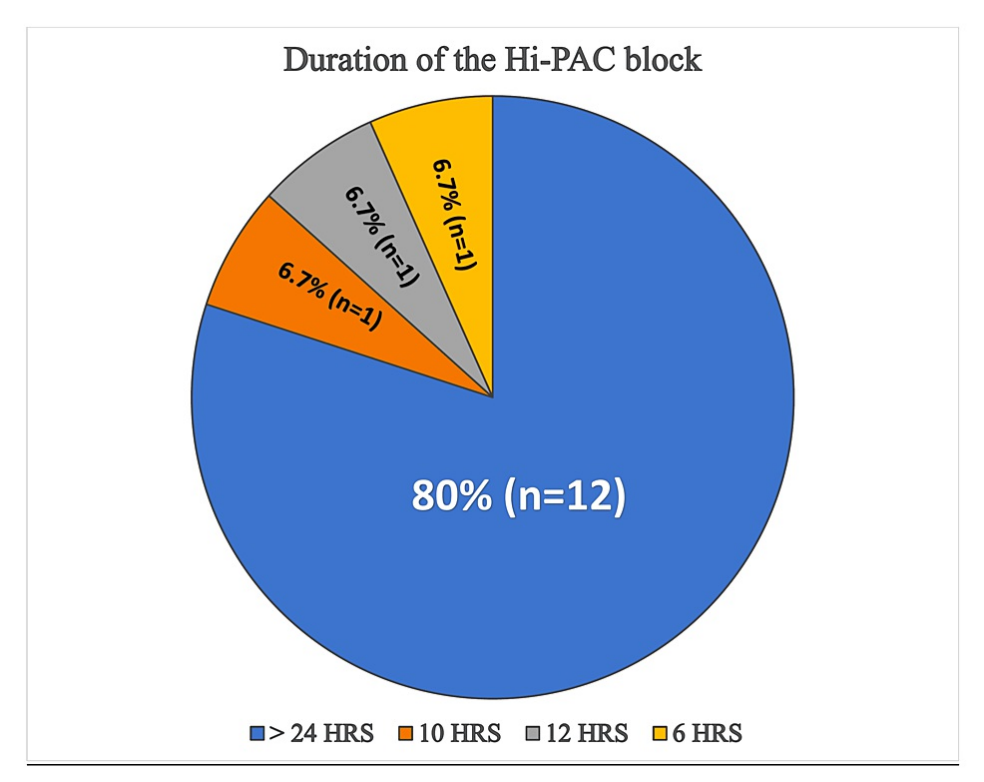
Pie diagram representing the total analgesic duration of the high-volume proximal adductor canal (Hi-PAC) block

Data were encoded and recorded in an MS Excel spreadsheet program (Microsoft® Corporation, Redmond, WA, USA). Statistical analysis was performed using SPSS version 23 (IBM Corporation, Armonk, NY, USA). Descriptive statistics were generated using means/standard deviations (SD) for continuous variables and frequencies and percentages for categorical variables (Table 1). Data were presented graphically using histograms/boxes for continuous data and bar charts/pie charts for categorical data (Figures 2-4).

**Table 1:** Demographic details and clinical characteristics of the study patients SD: standard deviation; SAB: subarachnoid block; CSEA: combined spinal and epidural anesthesia; Hi-PAC: high-volume proximal adductor canal; SN: saphenous nerve; FT: femoral triangle; NVM: nerve to vastus medialis

Clinical characteristics of the study patients	Mean ± SD/frequency (%)
Age (years)	51.20 ± 15.49
Age distribution	
≤40 years	4 (26.67%)
41-50 years	3 (20%)
51-60 years	4 (26.67%)
61-70 years	3 (20%)
>70 years	1 (6.70%)
Gender	
Male	11 (73.3%)
Female	4 (26.7%)
ASA grade	
I	8 (53.3%)
II	7 (46.7%)
Diagnosis	
Proximal tibia fracture	8 (53.3%)
Ankle fracture	7 (46.7%)
Type of neuraxial anesthesia	
SAB	8 (60%)
CSEA	5 (40%)
Duration of surgery (minute)	176.67 ± 45.30
Time to regain toe movements after neuraxial postoperatively (minute)	293.33 ± 75.96
Time to administer the Hi-PAC block postoperatively (minute)	5.13 ± 1.35
Drug around the popliteal sciatic nerve	
Could not scan	3 (20%)
Yes	11 (73.3%)
No	1 (6.7%)
Distance from the popliteal crease (cm)	8.47 ± 4.51
Drug around the SN in the FT	
Yes	15 (100%)
No	0 (0%)
Drug around the NVM in the FT	
Yes	14 (93.3%)
No	1 (6.7%)
Drug around the FN in the femoral crease	
Yes	0 (0%)
No	15 (100%)

We found no significant change in vital parameters before and after the Hi-PAC block (Figure 2). The mean postoperative pain scores at rest were 0.27 ± 0.704 immediately after the block (T0), 1.13 ± 1.767 six hours after the block (T6), 1.33 ± 1.718 twelve hours after the block (T12), and 1.20 ± 0.862 twenty-four hours after the block (T24). At the same time, the mean postoperative pain scores after squeezing the leg muscles were 0.60 ± 1.356 (T0), 1.20 ± 2.007 (T6), 2.00 ± 1.732 (T12), and 1.67 ± 0.816 (T24) (Figure 3).

The mean time to regain toe movements after the neuraxial block was 293.33 ± 75.96 minutes. The mean time to administer the Hi-PAC block was 5.13 ± 1.35 minutes. Post-block scanning distally into the popliteal region confirmed drug spread around the popliteal SCN in 11 patients with a mean distance from the popliteal crease of 8.467 ± 4.51 cm. However, due to an above-knee slab/cast, distal scanning could not be performed in three out of 15 patients. Post-block scanning proximally into the distal femoral triangle revealed drug spread around SN in 15 (100%) and nerve to vastus medialis (NVM) in 14 (93.3%) patients. However, none of the patients had drug distribution around the FN in proximal FT. The total duration of analgesia in most of the patients (12 out of 15) was >24 hours (Figure 4).

## Discussion

We found effective postoperative analgesia (VAS < 3) with motor-sparing advantage for sufficient duration (>24 hours) with the Hi-PAC block in below-knee surgeries. It helped reduce the need for rescue analgesics and subsequent opioid consumption postoperatively. Incorporating this novel RA technique into the postoperative pain management strategy resulted in increased patient compliance and satisfaction. Before deciding on an appropriate target-specific RA strategy, functional anatomy should always be considered. Hence, we followed the identify-select-combine approach [15]: identifying target innervations, selecting target nerve blocks, and combining selected blocks in a procedure-specific RA technique.

The innervation of the leg below the knee is not as complex as that of the knee joint. It involves the major contribution from the SCN and a minor contribution from the SN (on the medial side) [14]. The single-injection-dual-target technique of the Hi-PAC block targets the SN directly and the SCN indirectly [14]. Thus, it covers all procedure-specific innervations of the pain-generating components involved in below-knee surgeries. For the motor-sparing effect, we focused primarily on the involved dermatomes (of the incision site) and the osteotomes (of the surgical site), with the least focus on myotomes [14]. We administered the Hi-PAC block preferably after the beginning of the recovery of the motor movements from the neuraxial block. We also reassessed the motor effect of the block at certain intervals postoperatively. The prolonged duration of analgesia with the motor-sparing advantage was attributed to the timing of the block (after regaining toe movements) and the type/concentration of LA (0.1% ropivacaine) with the adjuvant (dexamethasone). The motor-sparing effect of the Hi-PAC block also aided in the postoperative assessment of patients for CS symptoms.

The pain caused by the impending CS is fundamentally disproportionate to the injury or surgery. It can be considered an earlier marker of ACS than the classic “six Ps” (pain, paresthesia, pallor, paralysis, poikilothermia, and pulselessness) [16]. Such a severe breakthrough postoperative pain out of proportion to the surgery demands additional analgesics regardless of an active RA technique. In this study, three of the 15 patients complained of moderate to severe pain (VAS > 6) within the expected duration (24 hours) after the Hi-PAC block. The increased analgesic demands in these patients necessitated rescue analgesia in the form of intravenous tramadol 100 mg postoperatively. Subsequent evaluations in these patients identified a tight slab as the cause of severe pain that was promptly removed by the surgical team, leaving all three patients comfortable and pain-free. The out of proportion pain in these patients suggested the involvement of ischemic and chemically mediated pain pathways apart from postsurgical inflammation. Such ischemic pain may not get blocked by any active RA technique [17].

In our study, such excruciating pain out of proportion to the surgical dissection provided timely warning signals indicating the development of increased compartment pressure. Thus, despite a well-functioning regional block, such ACS-pro conditions were promptly diagnosed and addressed simultaneously. This situation led us to recognize the importance of postoperative monitoring of the developing CS, particularly in high-risk patients/surgeries, without compromising effective postoperative analgesia. Therefore, regular postoperative monitoring of the clinical signs of ACS by trained personnel should be mandatory in below-knee surgeries. Selective sensory RA techniques with low concentrated LA are preferred to facilitate such monitoring. However, such postoperative monitoring should not depend on whether the RA technique is included or excluded in the MMA regimen.

In addition to postoperative monitoring, preoperative patient preparation and identification of possible risk factors are essential. The timing of surgery also plays an important role in preventing the development of postoperative ACS. Our surgical team thoroughly evaluated all study patients before posting them for surgery. Factors that caused the initial postponement of the surgery included significant edema over the affected area, blistering, the absence of skin wrinkling, or the inability to fold the skin over the affected area. These visual findings suggested increased compartment pressure due to injury and edema formation, which required preoperative monitoring in order to assess the further rise in compartment pressure or the need for emergency fasciotomy. Therefore, thorough preoperative and postoperative evaluations with the early suspicion of the disease help invoke an immediate response and prevent the future possibility of ACS [18].

**Figure 5:**
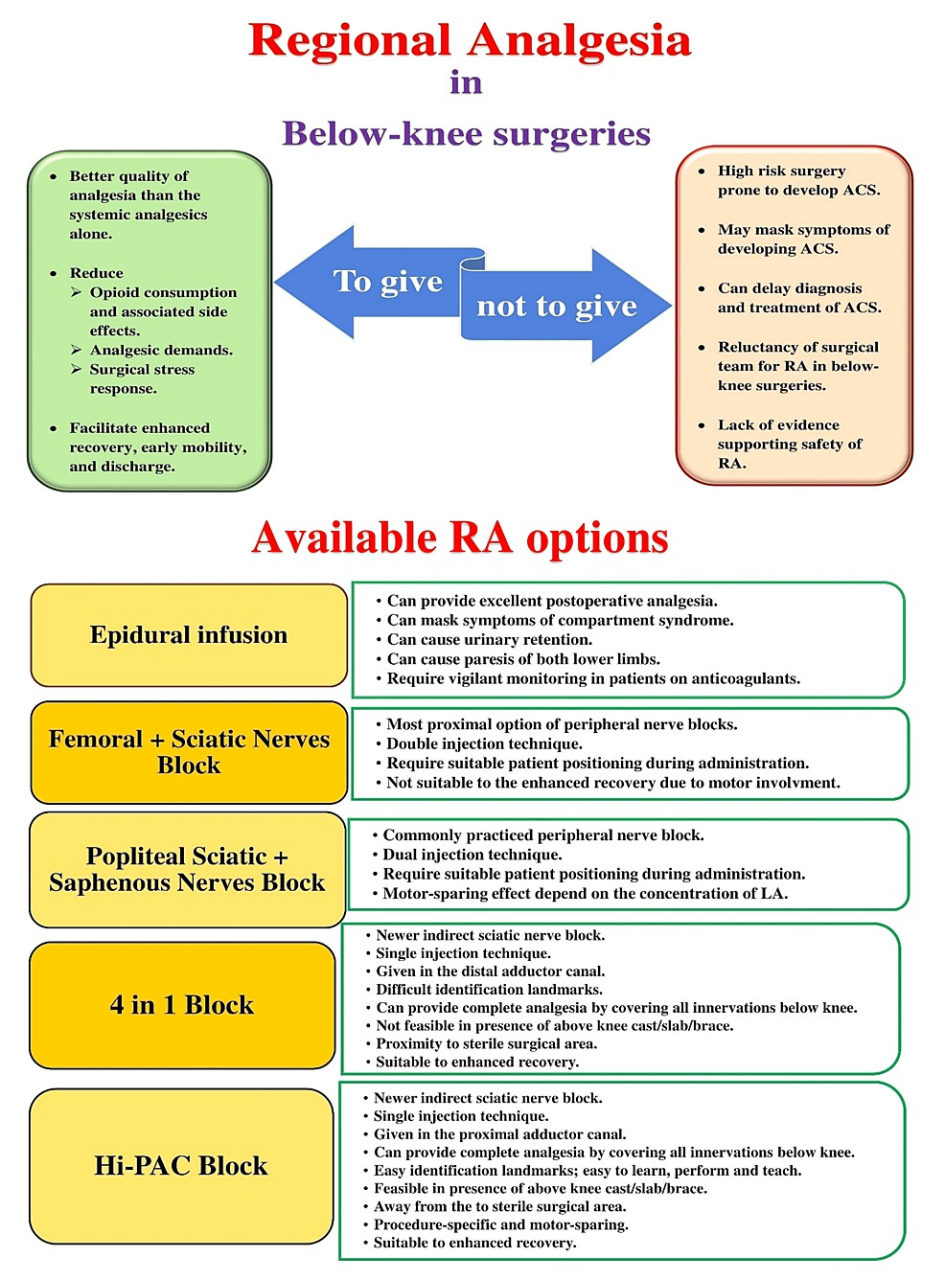
“To give or not to give” dilemma situation while considering regional analgesia for below-knee surgeries and available regional analgesia (RA) options ACS: acute compartment syndrome; RA: regional analgesia; LA: local anesthetic; Hi-PAC: high-volume proximal adductor canal

The use of RA in below-knee surgeries always creates a “to give or not to give” dilemma that supports both sides of the coin (Figure 5). It may be because of the misconception that RA can delay diagnosis and treatment of CS by masking the pain. We could not find any evidence suggesting a direct correlation of the RA with the development of CS postoperatively among described RA techniques (Figure 5). Many studies disputed the role of even dense RA technique in delaying ACS diagnosis [4]. Some studies recommended a diluted concentration of LA without using additives or the use of newer LA to avoid delaying ACS diagnosis [4,6,19-21]. However, the most reliable method for ACS diagnosis comprises frequent assessments of clinical status with monitoring of intracompartmental pressure (ICP) in high-risk patients [22]. Although ICP monitoring is considered the gold standard for ACS diagnosis, it is not always required. It can aid in diagnosis only if any uncertainty exists [23,24]. We believe that ACS diagnosis in the postoperative period depends solely on the regular vigilant assessment of the patient, especially in high-risk surgeries. The presence or absence of RA cannot prevent the development of ACS if it is going to develop. We could recognize the increasing compartment pressure in three patients complaining of severe out of proportion pain in the presence of an active regional block. It demonstrated the potential of the Hi-PAC block not to mask the symptoms and thus avoid delay in the diagnosis and treatment of developing CS. Also, among the various RA options available for below-knee surgeries (Figure 5), the Hi-PAC block outweighs all others due to its single-injection technique that covers all required innervations without changing the patient’s position and feasibility with an above-knee cast/slab/brace.

Every patient has the right to receive adequate, effective, and complete analgesia. As pain specialists, we also have a responsibility to keep our patients pain-free by selecting the most appropriate and safest analgesic protocol that must include RA as an essential ingredient. Incorporating the RA technique as an adjunct to MMA in any surgery has been proven multiple times to significantly reduce analgesic/opioid consumption and ultimately improve patient satisfaction by promoting enhanced recovery, early mobilization, and early discharge [25-28]. Such an inclusive approach helped us maintain our patients’ overall satisfaction score of 4/5 (very good) at discharge with the existing analgesia protocol.

This study was conducted as a pilot project for future comparative studies. The strengths of this study include recording the analgesic efficacy and motor-sparing effect of the Hi-PAC block for the first 24 hours, along with subsequent vigilant monitoring of the signs and symptoms of the developing compartment syndrome until discharge of all patients. The limitations include small sample size, monocentric nature, lack of a control group, and scope for various biases (selection and observation).

## Conclusions

In conclusion, the Hi-PAC block can provide effective postoperative analgesia without compromising the motor strength of the leg muscles and without masking the clinical symptoms of ACS during the periodic assessment of the patient. We believe that the Hi-PAC block can be an appropriate RA option to deal with ACS concerns/fear (ACS anxiety) associated with below-knee surgeries. Apart from all clinical advantages of the Hi-PAC block, it is a relatively easier block to learn (simple identification landmarks), administer (single injection with dual-target technique), overcome difficulties such as the above-knee cast, and teach to trainee regional anesthetists. This novel RA technique needs to be further explored with prospective multicenter clinical trials of larger sample sizes to substantiate the results better. In addition, it requires radiographic or cadaver dye studies to correlate clinical outcomes with neural component involvement.
